# Tumor cell-intrinsic and tumor microenvironmental conditions co-determine signaling by the glycoimmune checkpoint receptor Siglec-7

**DOI:** 10.1007/s00018-023-04816-6

**Published:** 2023-05-30

**Authors:** Eline J. H. van Houtum, Esther D. Kers-Rebel, Maaike W. Looman, Erik Hooijberg, Christian Büll, Daniel Granado, Lenneke A. M. Cornelissen, Gosse J. Adema

**Affiliations:** 1 Radiotherapy & OncoImmunology Laboratory, Department of Radiation Oncology, Radboud University Medical Center, Post 874, 6525 GA Nijmegen, The Netherlands; 2 Department of Pathology, The Netherlands Cancer Institute, Amsterdam, The Netherlands; 3 Department of Biomolecular Chemistry, Institute for Molecules and Materials, Radboud University Nijmegen, Nijmegen, The Netherlands

**Keywords:** Siglec-7, Immune checkpoint signaling, Sialic acid, Tumor microenvironment, Immunotherapy

## Abstract

Tumors create an immunosuppressive tumor microenvironment by altering protein expression, but also by changing their glycosylation status, like altered expression of sialoglycans. Sialoglycans are capped with sialic acid sugar residues and are recognized by Siglec immune receptors. Siglec-7 is an inhibitory immune receptor similar to PD-1, and is emerging as glycoimmune checkpoint exploited by cancer cells to evade the immune system. However, the exact cellular and molecular conditions required for Siglec-7-mediated immune cell inhibition remain largely unknown. Here, we report on the development of a chimeric Siglec-7 cell system that enables dissection of Siglec-7 signaling, rather than Siglec-7 binding. Antibody-induced clustering, sialic acid-containing polymers, and highly sialylated erythrocytes effectively induced Siglec-7 signaling, thereby validating functionality of this reporter system. Moreover, the system reveals tumor cell-dependent Siglec-7 signaling. Tumor-associated conditions important for Siglec-7 signaling were defined, such as Siglec-7 ligand expression levels, presence of the known Siglec-7 ligand CD43, and sialic acid availability for sialylation of glycans. Importantly, therapeutic targeting of the Siglec-7/sialic acid axis using a sialyltransferase inhibitor resulted in strong reduction of Siglec-7 signaling. In conclusion, using a newly established cellular tool, we defined a set of tumor-associated conditions that influence Siglec-7 signaling. Moreover, the system allows to assess the efficacy of novel cancer drugs interfering with the Siglec-7/sialic acid axis as immunotherapy to treat cancer.

**Supplementary Information:**

The online version contains supplementary material available at 10.1007/s00018-023-04816-6.

## Introduction

Tumors create an immune suppressive microenvironment, amongst others by upregulation of immune checkpoints [1]. Immune checkpoint therapy, such as PD-1 blockade, reactivates the anti-tumor immune response yielding a potent anti-tumor therapy in the clinic [2–4]. Unfortunately, many types of tumors do not respond adequately to immune checkpoint therapy [5]. A possible explanation for this might be the presence of other immune inhibitory mechanisms that prevent immune cell (re-)activation and prevent anti-tumor immunity independently of the targeted immune checkpoint receptor [5].

Tumor cells, next to abnormal protein expression, display an aberrant glycosylation profile, like increased sialic acid levels [6]. Glycosylation is a post-translational modification of proteins and lipids. Tumor glycosylation profiles have been associated with the acquisition of cancer hallmarks and do correlate with worse patient outcome in multiple tumor types [7, 8]. Sialoglycans form the natural ligands for the immune modulatory Siglec (sialic acid-binding immunoglobulin-like lectin) family [9, 10]. The human Siglec family consists of 14 receptors that are mainly expressed on immune cells [9, 11–14]. Siglecs were found to be upregulated on immune cells within the tumor microenvironment (TME), such as on CD8^+^ T cells and tumor associated macrophages [14–19]. Importantly, most of the Siglecs carry inhibitory ITIM and ITIM-like motifs that cause immune cell inhibition upon binding to their sialoglycan ligand [9, 11]. Siglec-mediated inhibition has been shown for several types of immune cells, such as natural killer (NK) cells and CD8^+^ T cells [12–14]. The structural resemblance of ITIM/ITIM-like-containing Siglecs to PD-1 in combination with expression of Siglecs on PD-1 positive immune cells, make Siglecs an interesting target for immune checkpoint blockade as potential cancer therapy for patients [11, 15, 20–22]. Interestingly, therapeutics that decrease sialic acid content in the TME as well as antibodies blocking sialic acid-Siglec interactions have been shown to improve tumor immunity in preclinical studies [23, 24].

Sialoglycan binding specificities of Siglecs have been identified with glycan arrays or glycan-displaying cells [25, 26]. Tumor-mediated Siglec signaling, however, is mostly indirectly assessed by either binding of recombinant Siglec Fc-proteins to tumor cells or immune cell function after tumor-immune co-cultures [11, 27, 28]. To what extent Siglec binding to sialoglycan ligands also induces signaling, has only been addressed in very few studies by investigating ITIM phosphorylation or SHP-1 recruitment [12, 29]. Clearly, there is a need for reporter systems that inform on Siglec signaling in a more robust and intermediate throughput manner. Moreover, little is known about tumor microenvironmental factors that could affect Siglec signaling, as for example hypoxia, metabolic conditions, or the presence of inflammatory factors that might influence sialoglycan structure and/or presentation [28]. Understanding Siglec signaling within the TME will be key to interfere with the Siglec/sialic acid axis in cancer. Therefore, a well-established model system to elucidate the molecular and cellular conditions to induce tumor-dependent Siglec signaling is highly desired [28].

Siglec-7 is expressed on multiple types of immune cells, such as NK cells, monocytes, macrophages and specific subsets of T cells [11]. It has been observed to influence immune cell function and multiple studies have associated expression of Siglec-7 or Siglec-7 ligands with poor patient survival [11, 13, 15, 30–34]. In the present study, we established a chimeric Siglec-7 receptor to directly assess tumor-dependent Siglec-7 signaling. Our data show that Siglec-7 signaling is dependent on various tumor-specific conditions, such as Siglec ligand levels, expression of the glycoprotein CD43 to present Siglec-7 ligand and sialic acid availability for glycan capping. This study highlights Siglec-7 signaling by TME components and implies that similar chimeric receptors could be used to study their preferred signaling conditions. Furthermore, the current chimeric Siglec-7 system could aid in investigating the potency of Siglec-7 targeting therapeutics as new immunotherapy agents for cancer treatment.

## Materials and methods

### Cell culture

Jurkat/MA cells were cultured in Iscove's Modified Dulbecco's Medium (IMDM, Gibco, 21,980,065), supplemented with 8% fetal bovine serum (FBS) (Sigma-Aldrich, F7524), 100 U/mL penicillin and 100 μg/mL streptomycin (Gibco, 15,140,163), and 500 μg/ml Hygromycin B (Invitrogen, 10,687,010) [35].

K562 cells (ATCC, CCL-243, RRID:CVCL_0004) were cultured in IMDM containing 10% FBS and 100 U/mL penicillin and 100 μg/mL streptomycin. T98G cells (ATCC, CRL-1690, RRID:CVCL_0556) were cultured in Dulbecco's Modified Eagle Medium (DMEM) with GlutaMAX (Gibco, 32,430,100) containing 10% FBS, 1% MEM non-essential amino acids (Gibco, 11,140,035), 1 mM sodium pyruvate (Gibco, 11,360,039), and 100 U/mL penicillin and 100 μg/mL streptomycin. IMR-32 cells (ATCC, CCL-127, RRID:CVCL_0346) were cultured in DMEM with GlutaMAX, supplemented with 10% FBS, 1% MEM nonessential amino acids, 1 mM sodium pyruvate, 55 µM β-mercaptoethanol (Gibco, 21,985,023), and 100 U/mL penicillin and 100 μg/mL streptomycin. SK-N-AS cells (ATCC, CRL-2137, RRID:CVCL_1700) were cultured in the same culture medium as IMR-32 cells, with the addition of 2 mM Glutamine (Lonza, BEBP17-605E).

HEK293-6E cells were a kind gift from H. Clausen (Copenhagen Center for Glycomics, University of Copenhagen, Copenhagen, Denmark) cultured in DMEM with GlutaMAX supplemented with 10% FBS and 100 U/mL penicillin and 100 μg/mL streptomycin. All cell lines were incubated in a humidified environment at 37 °C in 5% CO_2_. All cell lines were regularly tested for mycoplasma contamination using a mycoplasma detection test (Lonza, LT07-518).

Peripheral blood mononuclear cells (PBMCs) and erythrocytes were isolated from buffy coats from healthy donors obtained via Sanquin Blood Bank (Nijmegen, The Netherlands) using lymphoprep density gradient centrifugation (ELITech Group, AXI-1114547).

### Generation of chSiglec-7 and chSiglec-7R124A expressing Jurkat/MA cells

A gBlock gene fragment containing two extracellular amino acids, the transmembrane and the intracellular domains of the hCD3ζ open reading frame was obtained from Integrated DNA technologies and cloned into the pEGFP-N3 vector (Clontech), replacing EGFP. The extracellular domains of Siglec-7 and Siglec-7R124A were obtained using Q5 polymerase (New England Biolabs, M0491) mediated PCR amplification of a previously established pCI (Addgene, E1731) vector containing the codon-optimized Siglec-7 using the forward primer 5´-ACGACGCTCGAGACCGGTATGCTCTTGTTGCTGCTGCTGC-3´ and the reverse primer 5´-GTAGCAAAGCTTGGGCAGCAGCACGCCGGACACAG-3´. Then, the PCR products were cloned into the pN3 vector in front of the hCD3ζ open reading frame. Sequences were verified using Sanger sequencing.

Next, the obtained plasmids were linearized using restriction with PciI (New England Biolabs, R0655S) and purified using phenol/chloroform/isoamyl alcohol. Jurkat/MA cells were electroporated with the linearized plasmid. In short, 20 µg linearized plasmid was added to a 0.4 cm cuvette, cells were washed with Optimem (Gibco, 11,058,021) and 10 million cells in 200 µL Optimem were added to the cuvette. After a 3-min incubation at room temperature (RT), cells were electroporated with a Gene Pulser Xcell electroporation system, using an exponential protocol, 250 V and 950 μF. Cells were taken up in pre-warmed phenol red-free IMDM containing 8% FBS and incubated at 37 °C in 5% CO_2_ overnight. The next day, medium was changed to Jurkat/MA medium supplemented with 1 mg/ml geneticin (Gibco, 10,131,027).

After 3 weeks, cells were stained for Siglec-7 expression and Siglec-7 positive cells were single cell sorted using the FACS ARIA II SORP Flow Cytometer Cell Sorter (BD Biosciences). Flow cytometry was performed to assess expression of chSiglec-7 or chSiglec-7R124A over time.

### Flow cytometry

To determine Siglec-7 and CD43 membrane expression, cells were first washed with PBS and stained with eFluor 450 viability dye (eBiosience, 65–0863-18) (1:4000 in PBS) for 20 min at 4 °C and subsequently washed with PBS and subsequently with PBA (0.5% bovine serum albumin and 0.05% sodium azide in PBS). Next, cells were incubated for 20 min with PE-conjugated anti-Siglec-7 (clone 6–434, 2.5 μg/ml in PBA) (Biolegend, 339,204, RRID:AB_1501160) or APC-conjugated anti-CD43 (clone 10G7, 2 μg/ml in PBA) (Biolegend, 343,206, RRID:AB_2194072) at 4 °C. Cells were washed with PBA and subsequently acquired. For sorting cells, PBA without sodium azide was used in all described steps.

To determine Siglec-7 expression on immune cells, PBMCs were stained with eFluor 780 viability dye (eBioscience, 65–0865-18) (1:4000 in PBS) for 20 min at 4 °C, washed with PBS and stained for Siglec-7 as described above. Additionally, cells were stained with FITC-conjugated mouse anti-CD56 (clone NCAM16.2, 0.15 μg/ml in PBA) (BD Biosciences, 345,811, RRID:AB_2868832), PE-Cy7-conjugated mouse anti-CD3 (clone UCHT1, 1 μg/ml in PBA) (eBioscience, 25–0038-42, RRID:AB_1582253) and APC-conjugated mouse anti-CD14 (clone MφP-9, 1:100 in PBA) (BD Biosciences, 345,787, RRID:AB_2868813).

For recombinant human Siglec-7 Fc chimera binding, 0.4 μg/ml recombinant Siglec-7 Fc (R&D Systems, 1138-SL-050) or IgG1 Fc (R&D Systems, 110-HG-100) was pre-complexed for 20 min with 4 μg/ml Alexa Fluor 488-conjugated goat anti-human IgG (H + L, Invitrogen, A-11013, RRID:AB_141360) in Hank’s buffered salt solution (HBSS, Gibco, 14–025-092) at 4 °C. Cells were washed with PBS, stained for viability with eFluor 780 viability dye for 20 min at 4 °C and subsequently washed with PBS and subsequently with HBSS. Next, cells were stained with the pre-complexed recombinant human Siglec-7 Fc chimeras or IgG1 Fc for 40 min at 4 °C and washed with PBA before acquisition.

Fluorescence was measured using a BD FACS CantoII Flow Cytometer with the BD FACSdiva software (RRID:SCR_001456). Data were analyzed using the FlowJo software (version 10.7.0, Tree Star Inc., RRID:SCR_008520).

### Luciferase assays

During luciferase assays, cells were cultured in phenol red-free IMDM containing 0.5% FBS. Parental, chSiglec-7 and chSiglec-7R124A Jurkat/MA cells were stimulated with 10 ng/ml Phorbol 12-myristate 13-acetate (PMA, Invivogen, tlrl-pma) together with 1 µM ionomycin (Merck Millipore, I0634-1MG), 0 – 20 μg/ml Neu5Ac-polyacrylamide-biotin (GlycoNZ, 0035a-BP) or Neu5Gc-polyacrylamide-biotin polymers (GlycoNZ, 0018-BP) for 16 h.

For antibody-mediated Siglec-7 signaling, 96-well culture plates were coated with a concentration range (0 – 20 μg/ml) anti-Siglec-7 (clone S7.7 (Biolegend, 347,702) or clone QA79 (eBioscience, 14–5759-82)) or 20 μg/ml matched mIgG1 isotype control (clone MOPC-21, Biolegend, 400,102) for 3 h at 37 °C. Plates were extensively washed with PBS before addition of Jurkat/MA cells expressing either chSiglec-7 or chSiglec-7R124A for 16 h.

To examine signaling by chSiglec-7 upon co-culture with erythrocytes, erythrocytes were treated with sialidase by incubation with 200 mU/ml *C. perfringens* sialidase (Roche, 11,585,886,001) for 1 h at 37 °C and subsequently washed with IMDM culture medium containing 8% FBS. Next, Jurkat/MA cells expressing chSiglec-7 or chSiglec-7R124A and erythrocytes were washed with phenol red-free IMDM medium supplemented with 0.5% FBS. Subsequently, a co-culture was set up in a 1:2 ratio of Jurkat/MA cells and erythrocytes.

To assess signaling by chSiglec-7 upon co-culture by tumor cell lines, tumor cell lines were cultured for 3 days with 31.25 µM SiaFEt or DMSO. When required, IMR-32 cells were treated with 2 mM Ac_5_Neu5Ac for 3 days. At day 2, medium was refreshed, and cells were cultured for one additional day with 2 mM Ac_5_Neu5Ac. Next, cell lines were washed with phenol red-free IMDM medium supplemented with 0.5% FBS. Subsequently, co-cultures were started containing a 1:1 ratio of Jurkat/MA cells and T98G, SK-N-AS, K562, IMR-32 (parental or CD43^+^) or transfected HEK293-6E cells.

To minimize variation, Jurkat/MA cells were harvested to initiate above experiments at a density between 400,000 and 800,000 cells/mL. For all luciferase assays described, luminescence was assessed using the Bright-Glo™ Luciferase Assay System (Promega, E2620) according to the manufacturer’s protocol using a Victor3 multilabel plate reader (PerkinElmer). Background values of medium only controls were subtracted from the measurements.

### Fluorescence microscopy

Jurkat/MA cells were incubated on poly-L-lysine coated glass slides (Thermo Fisher Scientific, 631–1349) and allowed to adhere for 1 h at 4 °C. Glass slides were washed with PBS and subsequently fixed using 2% paraformaldehyde at RT. After 10 min, cells were washed with PBS and blocked with normal goat serum (1:50 in PBA, Jackson ImmunoResearch, 005–000-121) for 30 min at RT. Cells were subsequently incubated with 5 μg/ml anti-Siglec-7 (clone QA79, eBioScience) or a matched mIgG1 isotype control in PBA at 4 °C. After 45 min, cells were washed with PBS and incubated with 10 µg/ml Alexa Fluor 488 conjugated goat-anti-mouse IgG (H + L) in PBA at 4 °C. After 45 min, cells were washed with PBS and subsequently incubated with diamidino-2-phenylindole (2.5 μg/ml, Santa Cruz Biotechnology, sc-3598)) for 15 min at RT. Finally, cells were mounted using Fluoromount (Serva, 21,634.01).

To study clustering of chSiglec-7 upon co-culture with erythrocytes or K562 cells, they were treated with sialidase or SiaFEt, respectively, as described above. Next, K562 cells and erythrocytes were stained using the CellTrace Far Red Cell Proliferation kit (Invitrogen, C34564) by incubation for 20 min at 37 °C. After staining, cells were incubated with Jurkat/MA culture medium for 5 min at RT. Subsequently, the erythrocytes, K562 cells and Jurkat/MA cells expressing chSiglec-7 or chSiglec-7R124A were washed with 0.5% FBS containing IMDM culture medium, and a 16-h co-culture was set up as described above. Following the co-cultures, cells were transferred to poly-l-lysine coated glass slides and staining was performed as described above. Importantly, stainings containing K562 cells were additionally blocked with Fc block (1:5 in PBA) (Miltenyi Biotec, 130–059-901) for 15 min at 4 °C following the block with normal goat serum.

Images were acquired using a DM6000 microscope (Leica). Images were processed using ImageJ (NIH, RRID:SCR_003070).

### Western blot

Cells were washed with PBS and lysed in lysis buffer (1% SDS, 62.5 mM Tris–HCl pH 6.8, 1% Phenylmethylsulfonyl fluoride (v/v), containing 1 × protease inhibitor cocktail (Roche, 4,693,132,001) and 100 U benzonase nuclease (Novagen, 70,746–3). Subsequently, lysates were homogenized using a syringe, 4 × loading buffer was added (20% glycerol, 6% SDS, 25% Tris–HCl pH 6.8, 10% β-mercaptoethanol and bromophenol blue in MilliQ) and samples were boiled for 5 min at 95 °C. Lysates were resolved on a 7% SDS-PAGE gel and blotted to a nitrocellulose membrane. The membrane was blocked for 1 h at RT using blocking solution (3% bovine serum albumin (Roche, 10,735,094,001) and 1% non-fat dry milk (Santa Cruz Biotechnology, sc-2325) in PBS-T (0.1% Tween 20 in PBS)). Subsequently, the membrane was washed with PBS-T and stained using 1 μg/ml mouse anti-Siglec-7 (clone 194,211, R&D Systems, MAB11381) and 1:1000 rabbit anti-β-actin (clone 13E5, Cell Signaling, 4970S, RRID:AB_2223172) in 1:4 blocking solution in PBS-T overnight at 4 °C. After washing the blot with PBS-T, it was incubated with 0.2 μg/ml IRDye 800CW goat-anti-mouse IgG (Westburg, 926–32,210, RRID:AB_621842) and 0.2 μg/ml IRDye680RD goat-anti-rabbit (Westburg, 926–68,071, RRID:AB_10956166) for 1 h at RT. Finally, the blot was washed with PBS-T and imaged on an Odyssey CLx infrared imaging system (LI-COR).

### Generation of CD43^+^ HEK293-6E cells and CD43^+^ IMR-32 cells

The open reading frame of human CD43 was cloned from a pGEM-T vector with the human CD43 open reading frame (SinoBiological, HG13108-G) into the pEGFP-N3 vector, replacing EGFP. HEK293-6E cells were transfected with the CD43-pN3 plasmid using Metafectene (Biontex Laboratories GmbH, T040-2.0) as described by the manufacturer. CD43 expression was assessed after 24 h by flow cytometry as described above.

The pN3 plasmid containing the CD43 open reading frame was linearized by restriction with PciI (New England Biolabs) and purified using phenol/chloroform/isoamyl alcohol. Subsequently, 20 µg linearized plasmid was added to a 0.4 cm cuvette, IMR-32 cells were washed with Optimem and 10 million cells in 200 µL Optimem were added to the cuvette and incubated for 3 min at RT. Next, the cells were electroporated with the exponential protocol on the Gene Pulser Xcell electroporation system, using 200 V and 950 μF. The electroporated cells were taken up in pre-warmed phenol red-free RPMI containing 10% FBS and incubated at 37 °C in 5% CO_2_ overnight. Finally, the medium was changed to full IMR-32 medium as described above, supplemented with 0.6 mg/ml geneticin.

After approximately 5 weeks of culture with geneticin, cells were sorted for CD43 expression and collected as bulk. Finally, flow cytometry was performed as described above to verify CD43 expression.

### Statistical analysis

Statistical significance was determined using Prism 9.0.0 (GraphPad Software, RRID:SCR_002798). For comparison between groups a one-way ANOVA followed by Tukey’s multiple comparison test was performed. A p-value threshold of < 0.05 was used to determine significance (p < 0.05 *, p < 0.01 **, P < 0.001 ***, p < 0.0001 ****).

## Results

### Development of the chSiglec-7 signaling reporter system

**Fig. 1:**
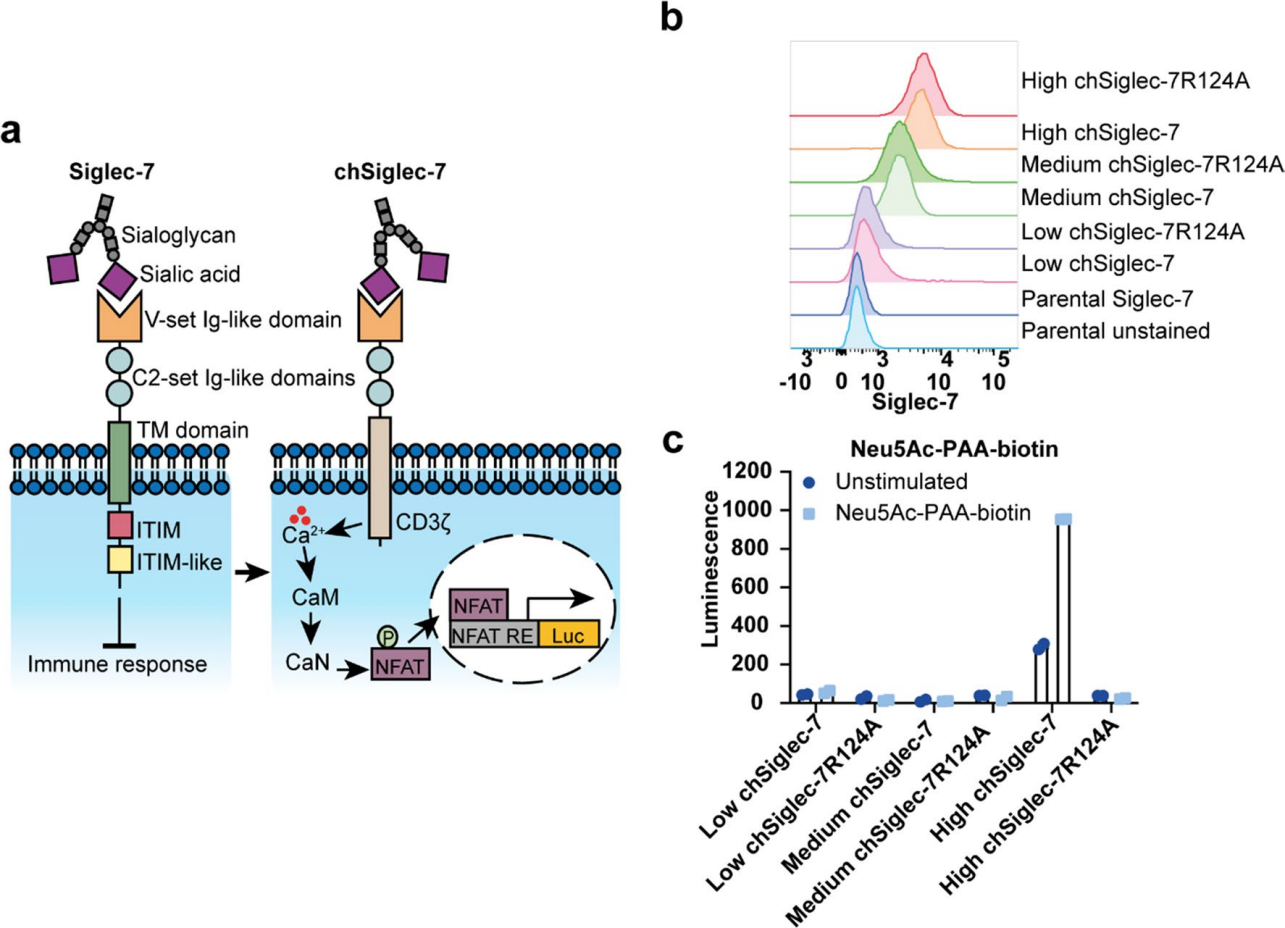
Development of the chSiglec-7 signaling reporter cells. **a** Schematic representation of the differences between Siglec-7 and chSiglec-7, in which the transmembrane (TM) and intracellular Siglec-7 domains are replaced by the CD3ζ chain. chSiglec-7 activation on Jurkat/MA cells leads to a calcium signaling pathway in which Calmodulin (CaM) and Calcineurin (CaN) are activated, resulting in NFAT translocation into the nucleus and subsequent binding to its response element (RE) and luciferase (Luc) transcription. **b** Siglec-7 membrane expression of parental Jurkat/MA cells and Jurkat/MA cells stably expressing low, medium and high levels of chSiglec-7 or chSiglec-7R124A was assessed by flow cytometry (n = 2 **c** signaling upon co-culture with sialic acid ligands was demonstrated by culture of the clones with Neu5Ac-PAA-biotin. Luminescence was assessed by luciferase assays. Bar diagrams are representative of two independent experiments and show mean ± SD

To study Siglec-7 signaling, we constructed a chimeric Siglec-7-hCD3ζ receptor (chSiglec-7). The extracellular domain of Siglec-7 was fused to the intracellular domain of human CD3ζ, its transmembrane domain and two extracellular amino acids to create chSiglec-7 (Fig. 1a) [36, 37]. Similarly, chimeric Siglec-7R124A (chSiglec-7R124A), in which the arginine 124 (R124) that is essential for sialic acid binding was mutated to alanine, was generated as control receptor [38, 39]. Jurkat/MA cells, a T cell line devoid of the T cell receptor β chain that contains an NFAT-luciferase reporter system, was selected to express the chSiglec-7 receptors (Fig. 1a) [35]. Upon chSiglec-7 signaling, intracellular calcium mobilization will lead to NFAT activation and eventually in transcription of the luciferase reporter gene (Fig. 1a).

First, we verified functionality of the NFAT reporter system in the parental Jurkat/MA cells using Phorbol 12-myristate 13-acetate (PMA) and ionomycin (Supplementary Fig. 1a). Moreover, we confirmed that the parental Jurkat/MA cell line showed no endogenous Siglec-7 expression (Fig. 1b and Supplementary Fig. 1b). Jurkat/MA cells were transfected with the chSiglec-7 or chSiglec-7R124A receptor and sorted as Siglec-7 + single cells. Three pairs of chSiglec-7 and chSiglec-7R124A Jurkat/MA clones with low, medium or high chSiglec-7 or chSiglec-7R124A expression were selected (Fig. 1b). PMA and ionomycin stimulation verified the presence of a functional NFAT activation reporter system in all clones (Supplementary Fig. 2a).

Next, we investigated the signaling capacity of the chSiglec-7 receptors in more detail. Plates coated with Siglec-7 antibody induced signaling by clustering of chSiglec-7 and chSiglec-7R124A cells (Supplementary Fig. 2b), but not in parental Jurkat/MA cells (Supplementary Fig. 1c). Subsequently, we investigated sialic acid-induced Siglec-7 signaling using a sialic acid (Neu5Ac) polymer (Fig. 1c). Polymer-induced Siglec-7 signaling was only detected in the cells expressing high levels of chSiglec-7. No signaling was observed for cells expressing the chSiglec-7R124A receptor, confirming sialic acid dependence. Cells expressing low/medium levels of chSiglec-7 were also not effectively activated by the polymers. An independent second clone expressing high chSiglec-7 levels verified activation of high chSiglec-7 by sialic acid polymer (Supplementary Fig. 3a-b). We therefore continued with the Jurkat/MA clones expressing high levels of chSiglec-7 or chSiglec-7R124A. Of note, the chSiglec-7 expression on the selected chSiglec-7 high Jurkat/MA clone is still well within the range of endogenous Siglec-7 expression levels as detected on immune cells isolated from blood of healthy donors. (Supplementary Fig. 4).

### Characterization of the high chSiglec-7 and chSiglec-7R124A clones

**Fig. 2:**
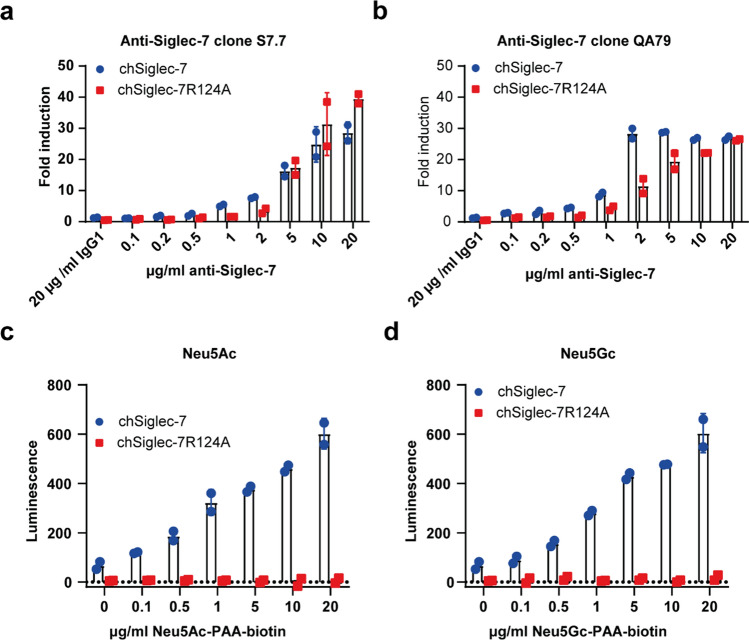
Characterization of the high chSiglec-7 Jurkat/MA cells by titrating coated antibody or soluble sialic acid polymer. **a** chSiglec-7 or chSiglec-7R124A on Jurkat/MA cells was crosslinked by plate coated anti-Siglec-7 clone S7.7, **b** anti-Siglec-7 clone QA79 or respective isotype control (n = 3). chSiglec-7 signaling was induced by culture with **c** 0–20 µg/ml Neu5Ac-PAA-biotin or **d** Neu5Gc-PAA-biotin polymer, (n = 3). Luminescence was measured using a luciferase assay. Bar diagrams are representative of three independent experiments and show mean ± SD

Characterization of the selected chSiglec-7 and chSiglec7R124A clones by western blot analysis and microscopy revealed equal chSiglec-7 and chSiglec-7R124A protein expression levels and cell membrane distribution, respectively (Supplementary Fig. 2c–e). To further explore the sensitivity of the model system, we cultured the chSiglec-7 and chSiglec-7R124A expressing Jurkat/MA cells in plates coated with different concentrations of anti-Siglec-7 monoclonal antibodies (Fig. 2a, b) or polymers decorated with Neu5Ac or Neu5Gc sialic acids (Fig. 2c, d). Signaling was induced with these stimuli in a concentration-dependent manner and no chSiglec-7R124A signaling could be detected at any of the polymer ligand concentrations, confirming sialic acid dependence.

### chSiglec-7 signaling is induced by sialic acid-carrying erythrocytes

**Fig. 3:**
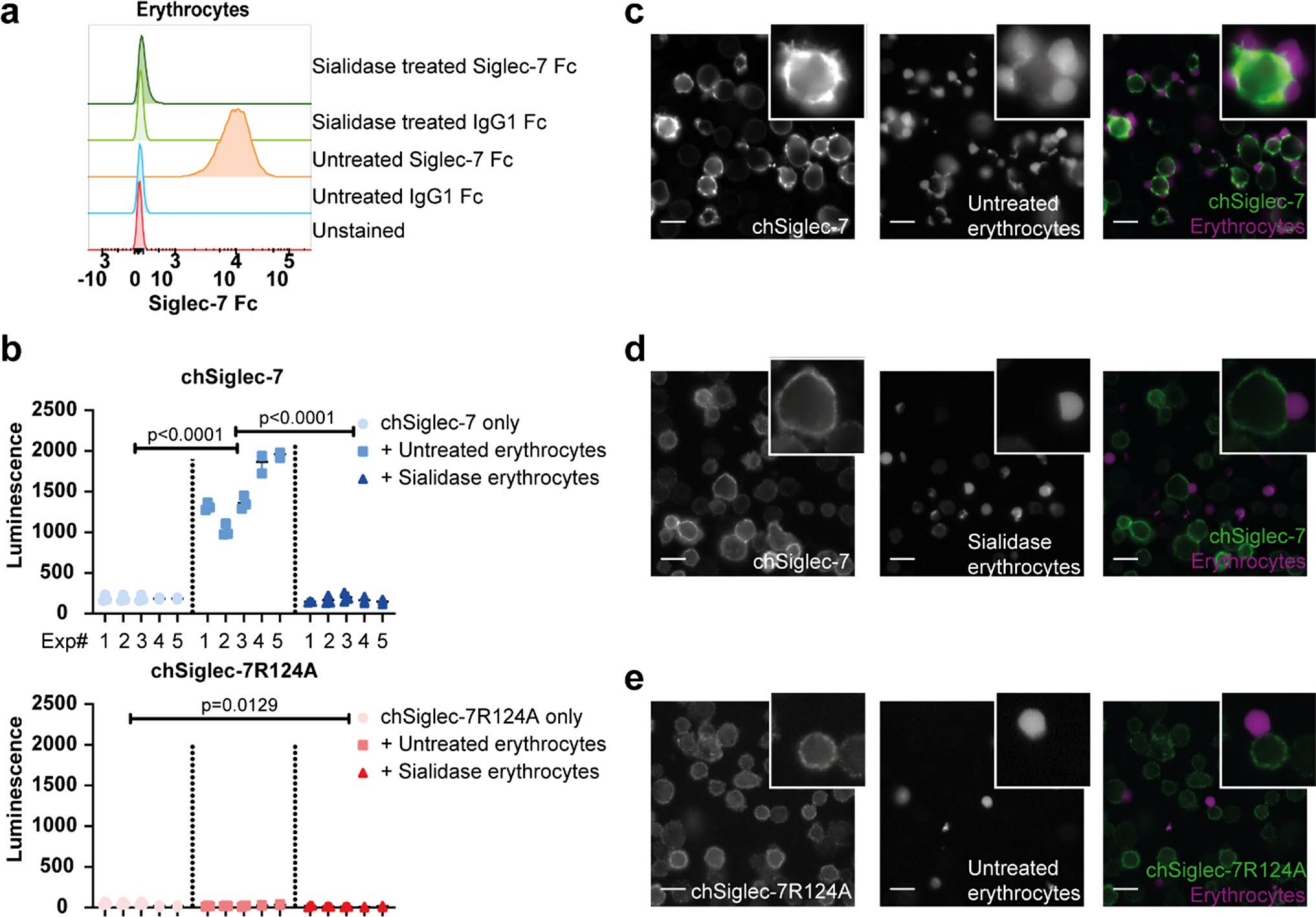
Erythrocytes induce chSiglec-7 signaling, accompanied by chSiglec-7 clustering. **a** Erythrocytes were either left untreated or treated with sialidase. Siglec-7 ligands were assessed by a recombinant human Siglec-7 Fc staining or respective IgG1 Fc control and measured by flow cytometry (n = 3). A representative figure of three independent experiments is shown. **b** chSiglec-7 or chSiglec-7R124A expressing Jurkat/MA cells were co-cultured with untreated or sialidase treated erythrocytes (1:2), after which luminescence was assessed using a luciferase assay, nested data of 5 donors are shown as mean ± SD. Figure represents nested data of 5 experiment, each containing data from one donor. **c** Erythrocytes were stained using Far Red Cell Trace and a co-culture was performed as described in (**b**). Subsequently, the cells were stained for Siglec-7 membrane expression (n = 2). **d** Same as in (**c**), but erythrocytes were treated with sialidase (n = 2). **e** Same as in (**c**), but chSiglec-7R124A expressing Jurkat/MA cells were co-cultured with untreated erythrocytes (n = 2). **c–e** Representative images are presented from two independent experiments. Scale bars: 25 µm. Signal in (**c–e**) was differently enhanced to visualize chSiglec-7 or chSiglec-7R124A distribution

Next, we investigated whether cells expressing natural Siglec-7 ligands were capable of eliciting chSiglec-7 signaling. Erythrocytes express high levels of Siglec-7 ligands, which can be completely removed from the cell surface by sialidase treatment (Fig. 3a) [40, 41]. Co-culturing erythrocytes with chSiglec-7 cells readily induced chSiglec-7 signaling, which was abrogated by sialidase treatment and absent in chSiglec-7R124A cells (Fig. 3b).

Subsequently, we determined the effect of erythrocyte co-culture on the membrane organization of the chSiglec receptors. Following the co-culture, the distribution of chSiglec-7 receptor was changed from an evenly distributed membranous pattern into a clustered pattern on the cell membrane (Fig. 3c). Importantly, the clusters concentrated at sites of cell contact between the chSiglec-7 cells and the erythrocytes. No clustering was detected when chSiglec-7 cells were co-cultured with sialidase treated erythrocytes or when using chSiglec-7R124A cells (Fig. 3d, e). From these experiments we concluded that signaling by chSiglec-7 can be induced by sialic acid expressing erythrocytes, which is accompanied by clustering of chSiglec-7 receptors on the membrane of Jurkat/MA cells.

### chSiglec-7 signaling is induced by sialic acid-bearing tumor cells

**Fig. 4:**
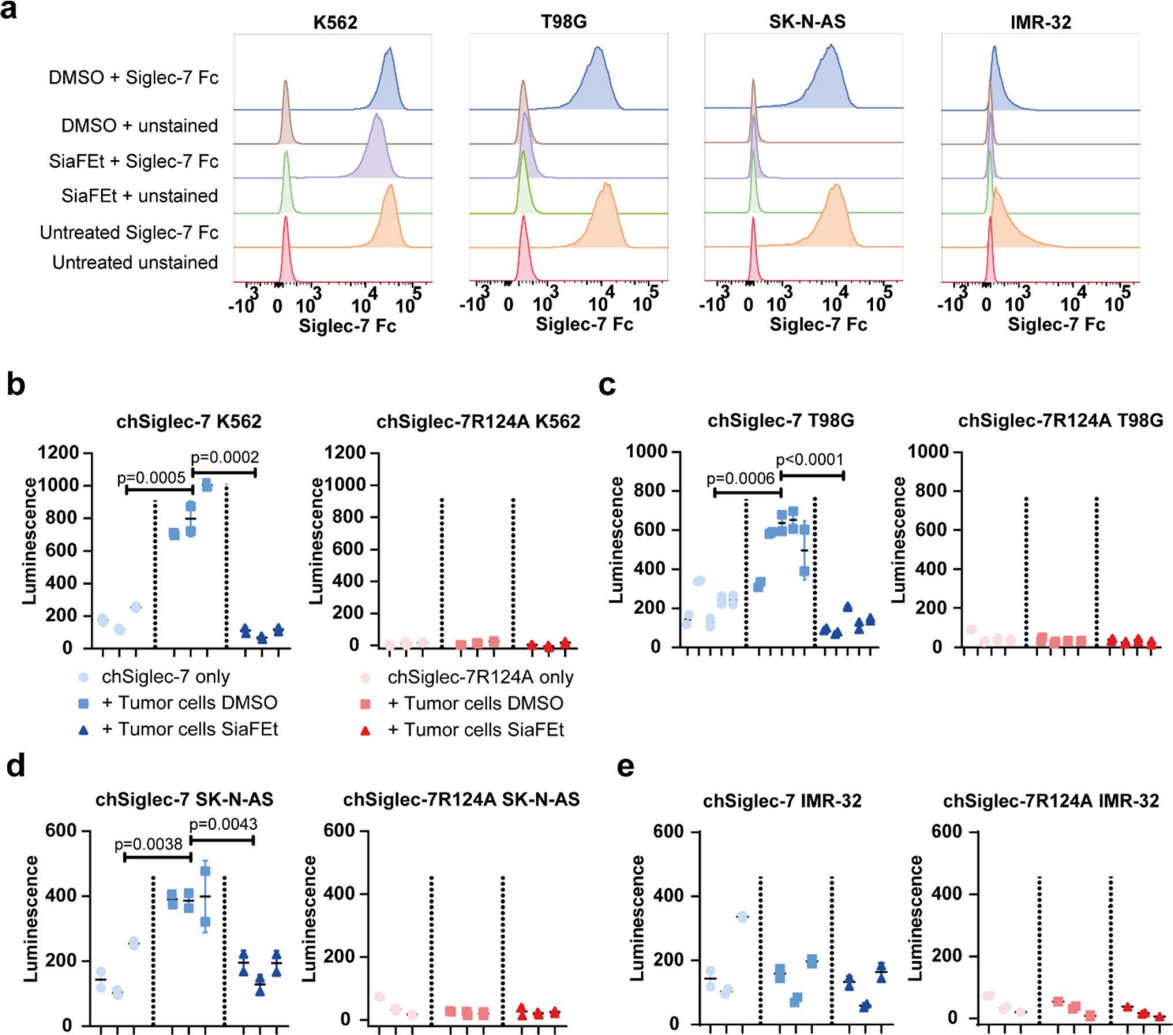
Tumor cells induce chSiglec-7 signaling, which is abolished by SiaFEt treatment. **a** T98G, SK-N-AS, K562 and IMR-32 cells were left untreated or were treated for 3 days with SiaFEt or DMSO as vehicle control. A recombinant human Siglec-7 Fc staining was performed to stain for Siglec-7 ligand expression. Staining was assessed by flow cytometry (n = 2). Representative data are shown from two independent experiments. **b** T98G, **c** SK-N-AS, **d** K562 and **e** IMR-32 tumor cells were treated for 3 days with SiaFEt or DMSO as vehicle control. chSiglec-7 (left panel) or chSiglec-7R124A (right panel) expressing Jurkat/MA cells were left unstimulated or co-cultured with these tumor cells (1:1). Luminescence was determined using a luciferase assay (n = 3–5). **b**–**e** Nested data are shown of 3 (K562, SK-N-AS, IMR-32) or 4 (T98G) experiments, each measured in duplicate

As many tumors show enhanced sialoglycan expression, we aimed to study Siglec-7 signaling upon tumor cell encounter. Hereto, three tumor cell lines expressing different Siglec-7 ligand levels were selected. The K562 chronic myelogenous leukemia cell line, the T98G glioblastoma cell line, and the SK-N-AS neuroblastoma cell line express high levels of Siglec-7 ligands, while the IMR-32 neuroblastoma cell line expresses much lower levels of Siglec-7 ligands as evidenced by flow cytometry analysis (Fig. 4a). To abolish Siglec-7 ligand expression and verify sialic acid dependence, tumor cells were depleted from sialic acids following treatment with the sialyltransferase inhibitor SiaFEt [42]. DMSO treatment served as vehicle control. We observed significant chSiglec-7 signaling upon co-culture with K562, T98G, and SK-N-AS cells but not in chSiglec-7R12A cells (Fig. 4b–d). Siglec-7 ligand dependence was further confirmed by the absence of signaling upon co-culture with IMR-32 and SiaFEt treatment of the tumor cell lines (Fig. 4b–e). T98G-induced Siglec-7 signaling was further confirmed using the second independent chSiglec-7 clone 2 (Supplementary Fig. 3b). In conclusion, the newly developed chSiglec-7 model system allows to determine the potency of tumor cells to induce Siglec-7 signaling and pre-treatment with the sialyltransferase inhibitor SiaFEt abolishes induction of Siglec-7 signaling by tumor cells.

### The chSiglec-7 receptor translocates to the site of contact with K562 tumor cells

**Fig. 5:**
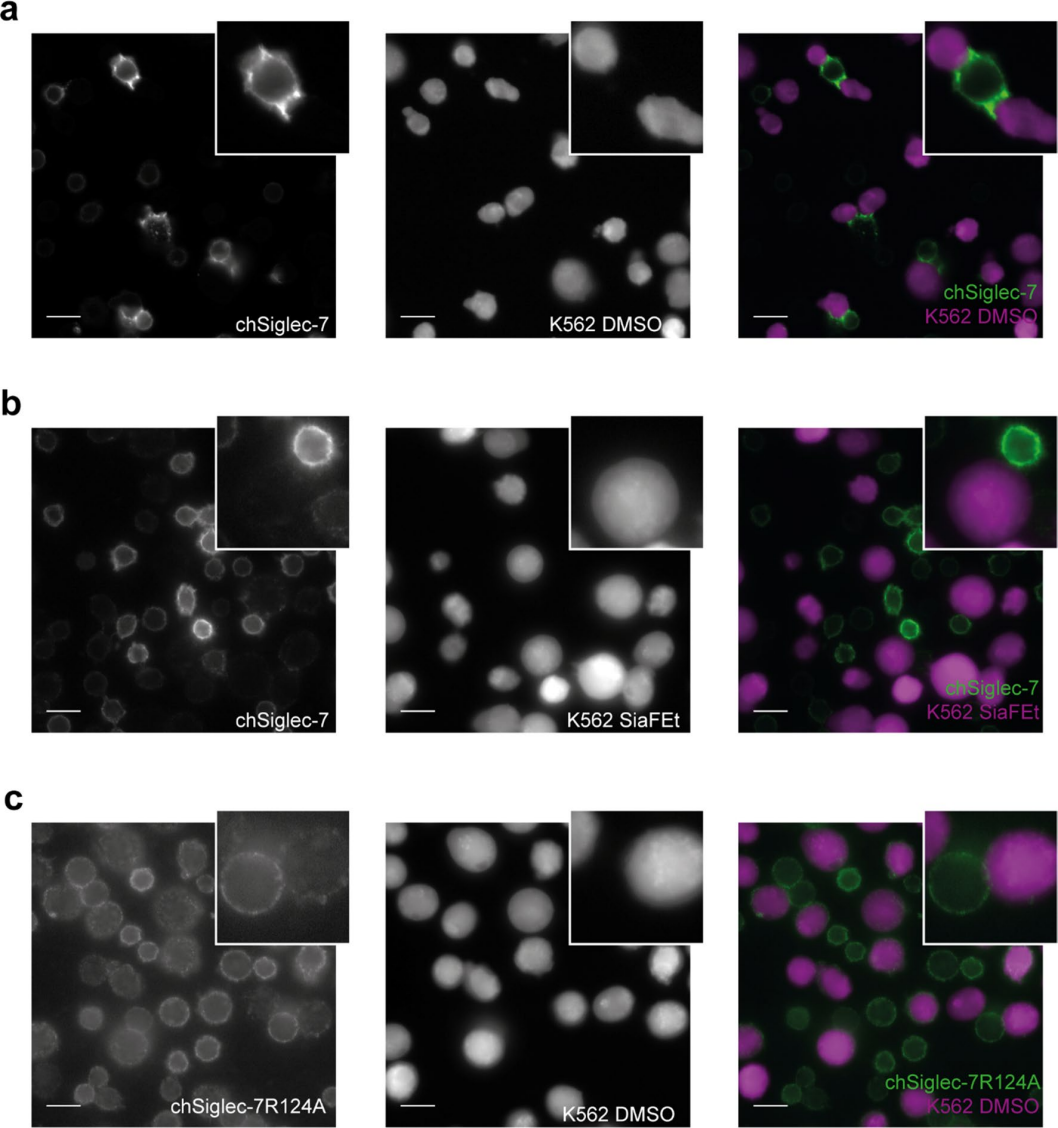
chSiglec-7 clusters at the site of contact with K562 tumor cells. chSiglec-7 expressing Jurkat/MA cells were co-cultured with K562 tumor cells that were treated for 3 days with **a** DMSO as vehicle control or **b** SiaFEt. **c** chSiglec-7R124A expressing Jurkat/MA cells were co-culture with K562 tumor cells that were treated for 3 days with DMSO. Before the co-culture, K562 cells were stained with a Far Red cell trace. Following the co-culture, cells were stained for Siglec-7 expression (n = 3). Representative images are shown from three independent experiments. Signal in each panel was differently enhanced to visualize chSiglec-7 or chSiglec-7R124A distribution. Scale bars: 25 µm

Recent literature has shown that Siglec-7 molecules on NK cells can relocate to the interaction site with K562 tumor cells [43]. To investigate whether a similar effect is seen using the chSiglec-7 receptor, the membrane expression pattern of chSiglec-7 was assessed upon co-culture with K562 cells or K562 cells lacking Siglec-7 ligands after SiaFEt treatment. Indeed, clustering of the chSiglec-7 receptor on Jurkat/MA cells was readily observed at the interaction site with K562 cells upon co-culture (Fig. 5a). No membrane chSiglec-7 clusters were formed when the chSiglec-7 Jurkat/MA cells were cultured with SiaFEt pretreated K562 cells (Fig. 5b) nor were chSiglec-7R124A clusters formed on the membrane when these were cultured with K562 cells (Fig. 5c). So, the chSiglec-7 receptors behave in a comparable manner as wild type Siglec-7 receptors co-cultured with K562 cells.

### A Siglec-7 ligand threshold is required to induce chSiglec-7 signaling

**Fig. 6:**
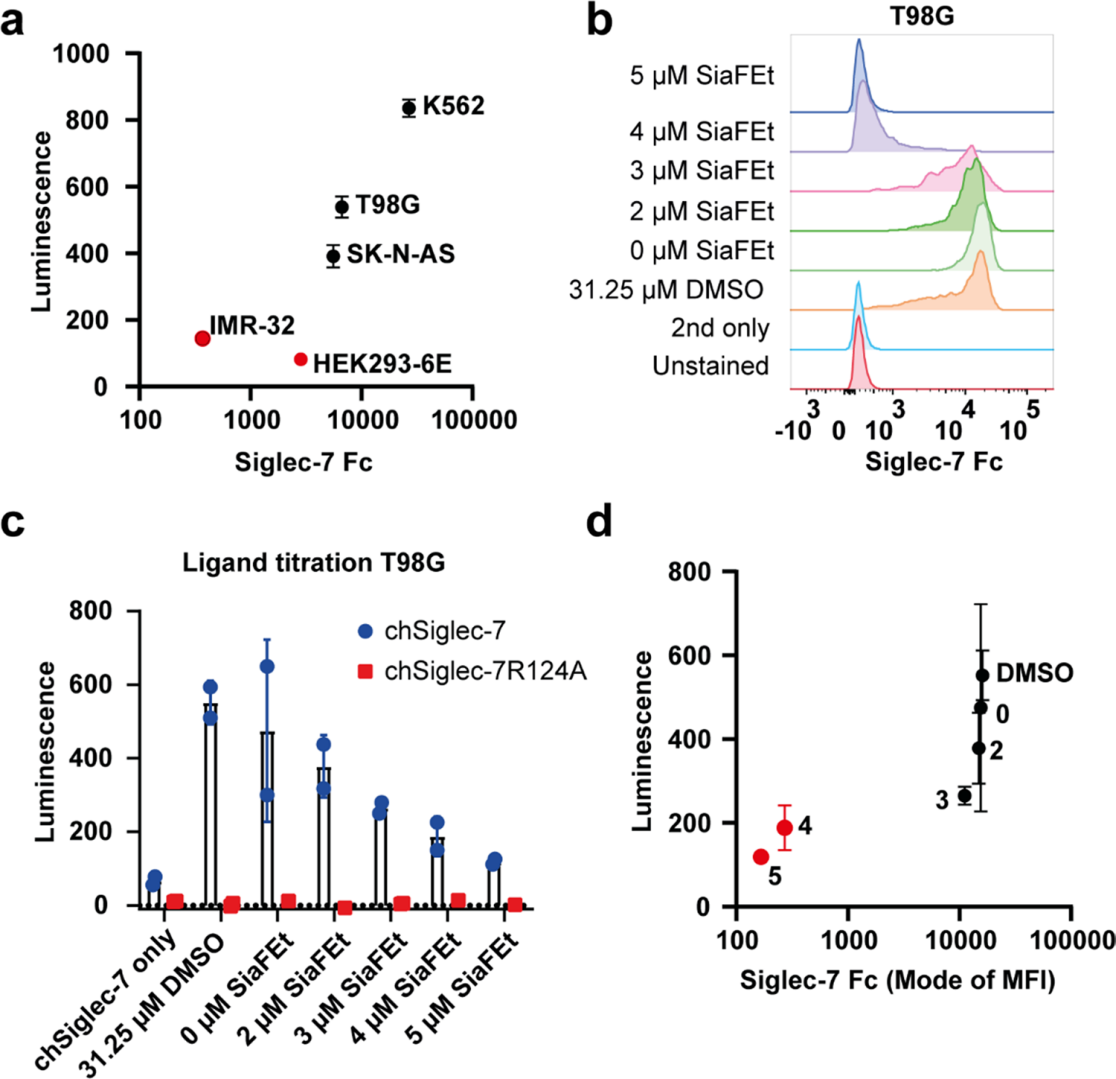
Siglec-7 Fc binding requires a threshold to induce chSiglec-7 signaling. **a** Siglec-7 Fc binding to cell lines was plotted against the luminescence value obtained from luciferase assays after co-culture with chSiglec-7 Jurkat/MA cells. Tumor cells capable of inducing chSiglec-7 signaling are depicted in black, tumor cells incapable of inducing Siglec-7 signaling are shown in red. **b** T98G cells were treated with different concentrations of SiaFEt or with DMSO as vehicle control for 3 days. A recombinant Siglec-7 Fc staining was performed to assess Siglec-7 ligand expression, which was measured by flow cytometry (n = 2). **c** chSiglec-7 Jurkat/MA cells were co-cultured with T98G cells treated with different concentrations of SiaFEt and a luciferase assay was performed to determine luminescence (n = 2). **d** Mode of Siglec-7 Fc staining on T98G cells treated with different concentrations SiaFEt (concentration in µM represented by the numbers within the graph) plotted against the luminescence value obtained after the co-culture of these cells with chSiglec-7 Jurkat/MA. Data are shown as mean ± SD. Representative data are shown from two independent experiments

The chSiglec-7 signaling strengths varied between the three tumor cell lines that were able to induce Siglec-7 signaling (Fig. 6a). Importantly, Siglec-7 Fc binding to the tumor cells did not correlate in a clear linear fashion with chSiglec-7 signaling that was induced upon co-culture with the tumor cells. Tumor cells with high Siglec-7 Fc binding were able to induce chSiglec-7 signaling, whereas co-culture with slightly lower Siglec-7 Fc binding (HEK293-6E and IMR-32) did not result in signaling. To further explore the relationship between Siglec-7 Fc binding and chSiglec-7 signaling, we titrated SiaFEt on T98G cells resulting in gradually decreasing sialic acid expression levels on the T98G cells. Only a small shift in Siglec-7 ligand expression (Fig. 6b) resulted in a sharp decrease in signaling by chSiglec-7 (Fig. 6c, d). These results strongly suggest that a certain Siglec-7 ligand threshold on tumor cells is required to induce chSiglec-7 signaling. Altogether, the chSiglec-7 signaling system provided insight in the extent to which Siglec-7 ligand expression influences chSiglec-7 signaling.

### Sialic acid capping determines CD43-mediated chSiglec-7 signaling

**Fig. 7:**
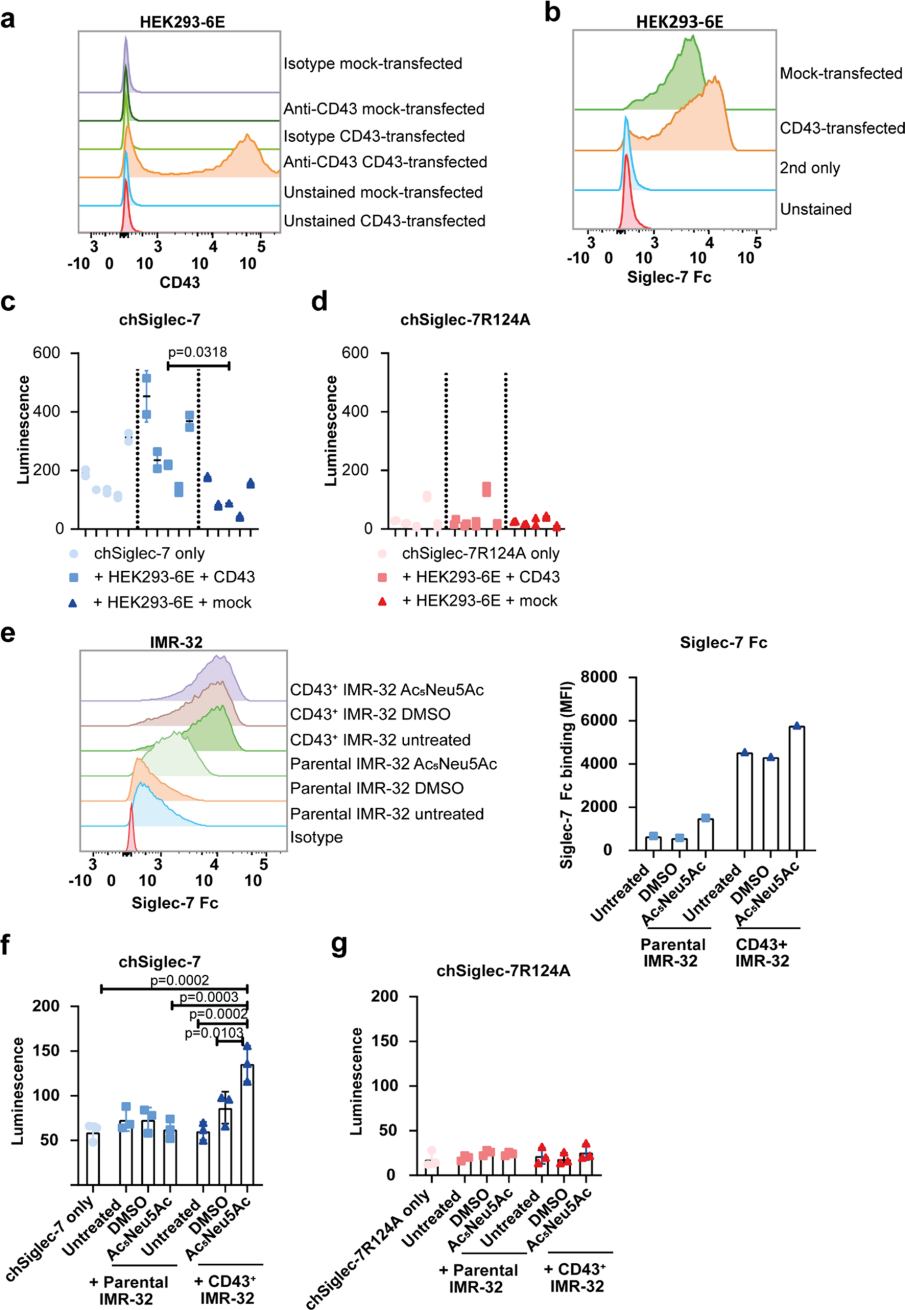
CD43-expressing cells can induce chSiglec-7 signaling under the condition of sufficient sialic acid availability. **a** HEK293-6E cells were transfected to express CD43 or were mock-transfected and CD43 expression was determined by flow cytometry and compared to the respective isotype control. **b** Siglec-7 ligand expression was determined by a recombinant human Siglec-7 Fc staining, measured by flow cytometry. **c** chSiglec-7 or **d** chSiglec-7R124A expressing Jurkat/MA cells were co-cultured (1:1) with the CD43 or mock transfected HEK293-6E cells and luminescence was assessed using a luciferase assay (n = 5). **c**, **d** Nested data are presented of 4 experiments, each performed in duplicate. **e** Siglec-7 Fc binding to parental IMR-32 cells or CD43^+^ IMR-32 cells was assessed by flow cytometry and the MFI was quantified. **f** chSiglec-7 or **g** chSiglec-7R124A expressing Jurkat/MA cells were co-cultured (1:1) with parental IMR-32 cells or CD43^+^ IMR-32 cells and luminescence as assessed using a luciferase assay (n = 2). IMR-32 cells were pretreated with Ac_5_Neu5Ac or DMSO as vehicle control for 3 days, which was refreshed on day 2. Representative data are shown of two independent experiments and data present mean ± SD

To start defining conditions affecting Siglec-7 signaling, we determined the impact of glycoprotein expression and sialic acid availability in our chSiglec-7 model system. A potent Siglec-7 ligand described in literature is CD43, a densely O-glycosylated membrane glycoprotein [25, 43, 44]. To find out whether expression of CD43 will enable cells with low endogenous Siglec ligand expression to induce Siglec-7 signaling, we transiently transfected HEK293-6E cells to express CD43 (Fig. 7a). Indeed, expression of CD43 enhanced binding of recombinant human Siglec-7 Fc to the HEK293-6E cells and induced chSiglec-7 signaling when compared to mock-transfection (Fig. 7b–d).

Finally, we investigated whether tumor cells normally incapable of inducing chSiglec-7 signaling, could gain the capacity to trigger chSiglec-7 signaling upon CD43 expression. CD43-expressing IMR-32 cells (Supplementary Fig. 5) enhanced Siglec-7 Fc binding compared to parental IMR-32 cells (Fig. 7e**)**, but were not able to induce chSiglec-7 signaling (Fig. 7f, g). Again, showing that even high Siglec-7 Fc binding does not directly imply chSiglec-7 signaling. We hypothesized that additional sialic acid is required for CD43^+^ IMR-32 cells to induce chSiglec-7 signaling. Sialic acid analog Ac_5_Neu5Ac supplementation caused a small increase in Siglec-7 Fc binding to both parental IMR-32 cells and CD43^+^ IMR-32 cells (Fig. 7e) [45]. Interestingly, only CD43^+^ IMR-32 cells supplemented with Ac_5_Neu5Ac gained the capacity to induce chSiglec-7 signaling. This signaling was fully sialic acid dependent as no activation of the mutated chSiglec-7R124A was observed (Fig. 7f, g). In conclusion, CD43 expression by IMR-32 tumor cells can induce chSiglec-7 signaling, but only in the presence of sufficient sialic acid availability.

Together, we found that a combination of tumor-intrinsic factors, such as Siglec ligand expression, and tumor microenvironmental factors, such as sialic acid availability co-determine Siglec-7 signaling.

## Discussion

The Siglec/sialic acid axis forms an immune escape route in tumors expressing high levels of sialoglycans, thus potentially forming a novel target for cancer immunotherapy [6, 14–20, 28]. However, many aspects regarding the interaction between Siglecs and tumor-associated sialoglycans and especially the subsequent Siglec signaling remain poorly studied. Here, we have developed a model system to enable assessment of Siglec-7 signaling. Functionality thereof was verified using antibody-mediated clustering and by culture with sialic acid polymers or sialylated erythrocytes. Interestingly, we demonstrated tumor-mediated Siglec-7 signaling that is accompanied by membrane clustering of the chSiglec-7 receptor. Therapeutic potential of the sialyltransferase inhibitor SiaFEt was demonstrated, as tumor cell treatment with the inhibitor completely abolished Siglec-7 signaling. Importantly, we started identifying tumor-intrinsic and tumor microenvironmental conditions that affect Siglec-7 signaling, such as Siglec ligand levels, CD43 expression, and sialic acid availability in the TME.

First of all, when studying the relationship between Siglec-7 Fc binding and chSiglec-7 signaling, we showed that only high Siglec-7 ligand levels were able to generate robust chSiglec-7 signaling, whereas low levels of Siglec-7 ligands did not result in chSiglec-7 signaling. Interestingly, this is in line with research by Hudak et al. (2014), which also hinted towards a discrepancy between recombinant Siglec-7 Fc binding patterns to sialoside glycopolymers and subsequent immune modulation [12]. In their study binding patterns of recombinant Siglec-7 Fc to different glycopolymers did not correlate well with the measured NK cell cytotoxicity to cells presenting these glycopolymers. Importantly, the mutant chSiglec-7R124A did not signal by any of the used stimuli, despite the recent discovery of a second sialic acid binding region in Siglec-7, which remained wild type in our chSiglec-7R124A mutant [46]. Moreover, presence of the glycoprotein Siglec-7 ligand CD43 alone was not sufficient for IMR-32 tumor cells to induce chSiglec-7 signaling, but presumably required sufficient sialic acid availability for sialylation. These results illustrate the complex establishment of Siglec-7 signaling, in which tumor-intrinsic and tumor microenvironmental conditions play a role.

Siglecs have previously been identified to interact with erythrocyte-associated sialoglycans and here we have successfully used our Siglec-7 signaling model system to verify Siglec-7 signaling induced by sialoglycans on erythrocytes [40, 41]. Functionally, we hypothesize that erythrocyte-induced Siglec-7 signaling prevents immune cell activation and associated inflammation in the bloodstream. Likewise, it has previously been hypothesized that Siglec-7 on eosinophils prevents eosinophil activation and Siglec-9 on neutrophils has been shown to prevent neutrophil activation upon binding to erythrocytes-associated sialoglycoproteins [41, 47]. However, more extensive research is required to elucidate the physiological function of erythrocyte-mediated Siglec-7 signaling.

We have shown here that sialic acid polymer-induced signaling was only observed by reporter cells with high expression of chSiglec-7, but not by cells with low/medium expression levels of chSiglec-7. Therefore, future research should further elucidate the relevance of membrane expression levels and organization of Siglec-7 for establishing signaling domains and what TME factors affect this. Such research could identify strategies to interfere with the Siglec/sialic acid axis in the TME.

CD43 contains a mucin-like domain that is heavily O-glycosylated and was previously found to function as Siglec-7 ligand on K562 tumor cells [43, 44]. Similar to other Siglec glycoprotein ligands such as MUC1, GP1bα or LGALS3BP, CD43 has many glycosylation sites for glycan presentation in a multivalent manner [25, 28, 48–50]. Importantly, expression of such Siglec ligands can be enhanced within the TME and might therefore be interesting targets for immunotherapy [28]. Studying Siglec signaling induced by multivalent glycoproteins can reveal the importance of multivalence for Siglec signaling. We here observed that overexpression of CD43 in IMR-32 tumor cells can induce Siglec-7 signaling under the condition of sufficient sialic acid availability. For HEK293-6E cells, CD43 expression was sufficient to induce chSiglec-7 signaling and no additional sialic acid supplementation was required. We hypothesize that the untreated CD43^+^ IMR-32 cells are suboptimally sialylated, and that additional sialic acid increases the level of sialylation measured by Siglec-7 Fc binding. However, the impact of the overall composition of the CD43-associated sialoglycans remains unknown. CD43-induced Siglec-7 signaling might be dependent on the expression of specific glycans, as it has been shown for Siglec-7 Fc binding to CD43 upon expression of disialyl-T [25]. Importantly, a change in O-glycosylation profile is often observed in tumors, which might affect CD43 glycosylation and thereby perhaps also CD43-mediated Siglec-7 signaling in the TME [51]. These speculations on overall glycosylation profile, however, warrant further investigation. Altogether, the current findings illustrate the influence of the TME on the capability of tumor cells to induce Siglec-7 signaling.

We have shown here that treatment of tumor cell lines with the sialic acid inhibitor SiaFEt abolished all chSiglec-7 signaling. Interestingly, we have previously demonstrated therapeutic efficacy of a similar sialic acid mimetic in vivo [23]. Intratumoral injections with Ac_5_3Fa_x_Neu5Ac resulted in reduced tumor growth, enhanced infiltration of CD8^+^ T cells and NK cells, reduced infiltration of regulatory immune cells leading to enhanced tumor cell killing by CD8^+^ T cells. Furthermore, a reduced sialic acid expression was observed in tumor cells, therefore it would be interesting to investigate the influence of this therapeutic on Siglec-7 signaling in vivo using our chSiglec-7 signaling model system.

In conclusion, we have shown the influence of specific tumor cell-intrinsic and tumor microenvironmental conditions on Siglec-7 signaling and our new Siglec-7 signaling model system can now pave the way to study other remaining knowledge gaps regarding tumor-mediated Siglec-7 signaling. The influence of tumor microenvironmental factors on Siglec-7 signaling that can now be investigated are, for instance, the effect of cell-bound and secreted tumor sialoglycans, the effect of hypoxia, a change in metabolism, and the presentation of Siglec-7 protein and lipid ligands on TME resident cells, including tumor-associated fibroblasts. Besides increasing our fundamental understanding on Siglec signaling, this model will be of translational value as it allows screening of therapeutics targeting the Siglec-7/sialic acid axis to prevent immune cell inhibition.

### Supplementary Information

Below is the link to the electronic supplementary material.

Supplementary file1 (DOCX 1673 KB)

## Data Availability

Data generated during the current study are available upon reasonable request.
