# Supplementary material for: Tumor cell-intrinsic and tumor microenvironmental conditions co-determine signaling by the glycoimmune checkpoint receptor Siglec-7
Source: Cell Mol Life Sci. 2023 May 30;80(6):169. doi: 10.1007/s00018-023-04816-6 (PMC10229725; doi:10.1007/s00018-023-04816-6)
Supplement: Supplementary file 1 — Supplementary file1 (DOCX 1673 KB) [file 18_2023_4816_MOESM1_ESM.docx]

**Supplementary figures**

**Tumor cell-intrinsic and tumor microenvironmental conditions co-determine signaling by the glycoimmune checkpoint receptor Siglec-7**

Eline J.H. van Houtum^1^, Esther D. Kers-Rebel^1^, Maaike W. Looman^1^, Erik Hooijberg^2^, Christian Büll^3^, Daniel Granado^1^, Lenneke A.M. Cornelissen^1^, Gosse J. Adema^1^


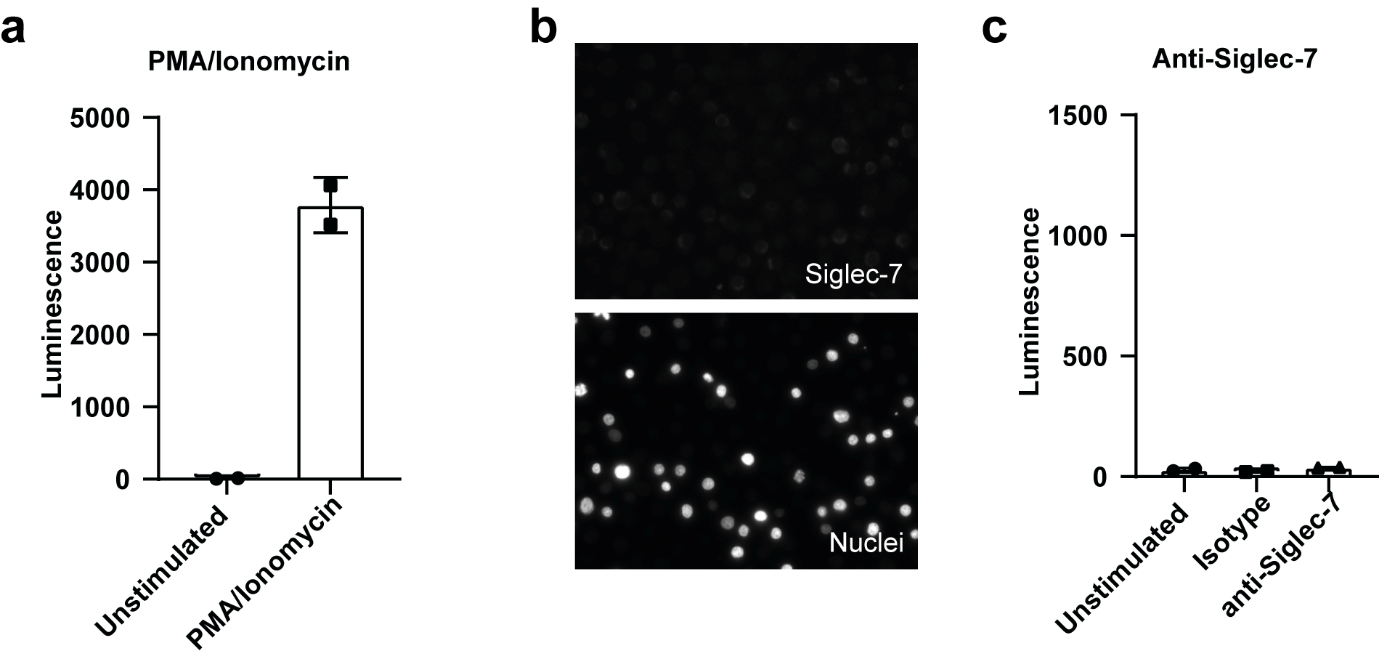


**Supplementary Fig. 1** Verification of functionality and absence of Siglec-7 expression/signaling in parental Jurkat/MA cells. **(a).** Parental Jurkat/MA cells were stimulated with PMA and ionomycin to activate the NFAT reporter system and luminescence was measured using a luciferase assay. **(b).** Membrane staining of Siglec-7 by parental Jurkat/MA cells was investigated using an immunofluorescent staining. **(c).** Cells were cultured in wells coated with anti-Siglec-7 antibody and luminescence was assessed using a luciferase assay. Representative data of two independent experiments are shown and bar diagrams present mean ± SD.


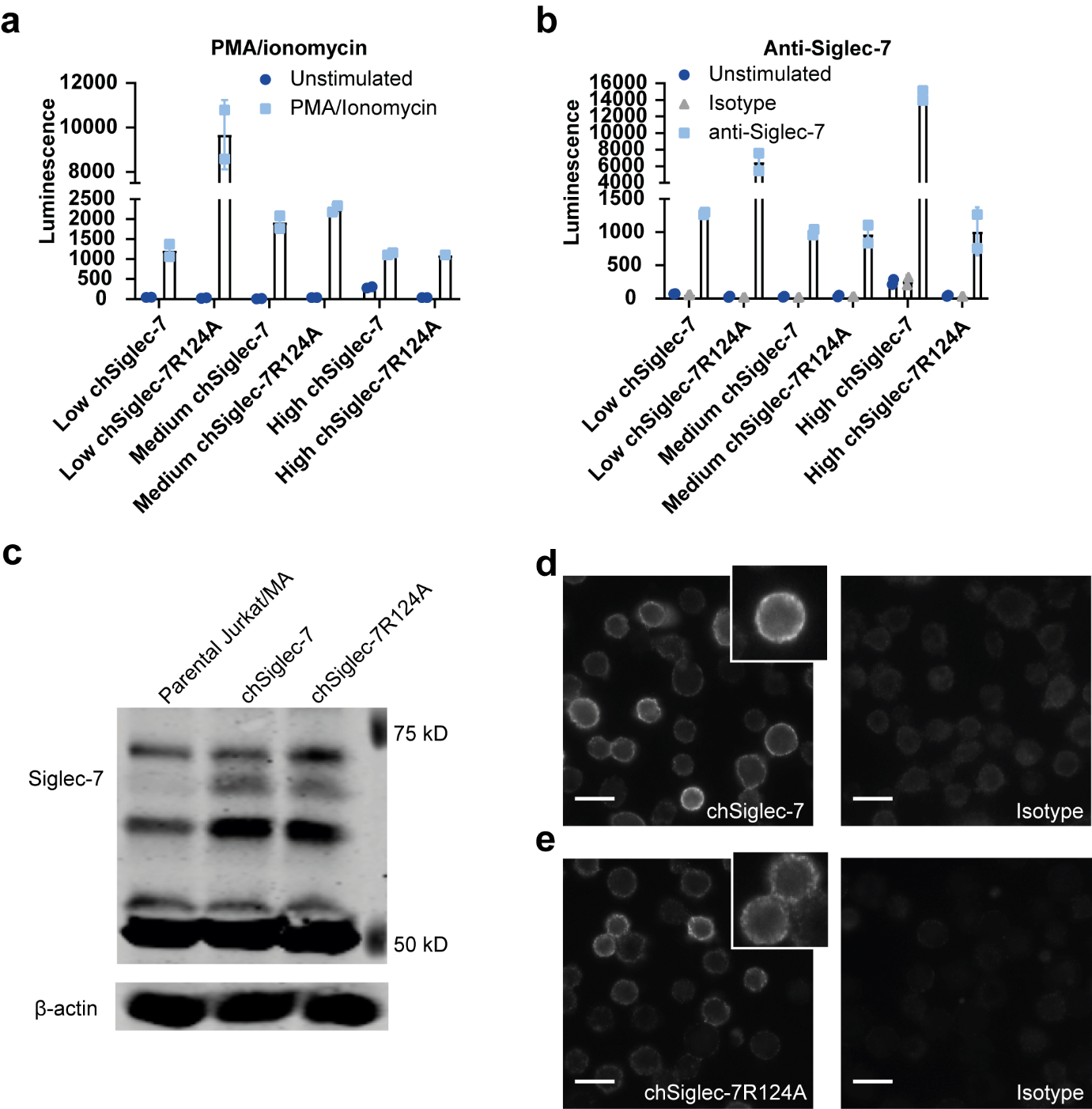


**Supplementary Fig. 2** Characterization of the chSiglec-7 and chSiglec-7R124A clones. (**a**) Functionality of the NFAT reporter system of the different clones was verified by stimulation by incubation with PMA and ionomycin. (**b**) NFAT signaling upon Siglec-7 crosslinking was shown by culturing the cells in wells coated with anti-Siglec-7 antibody or isotype control. Luminescence was measured with a luciferase assay. Bar diagrams are representative of three independent experiments and present mean ± SD. (**c**) Total Siglec-7 protein expression in parental Jurkat/MA cells or the high clones expressing chSiglec-7 or chSiglec-7R was assessed by western blot (n=3). Immunofluorescent stainings were performed to assess membrane expression patterns of (**d**) chSiglec-7 or (**e**) chSiglec-7R124A (n=3), scale bars: 25 µm. Signal in (d) and (e) panel was differently enhanced to visualize chSiglec-7 or chSiglec-7R124A distribution.


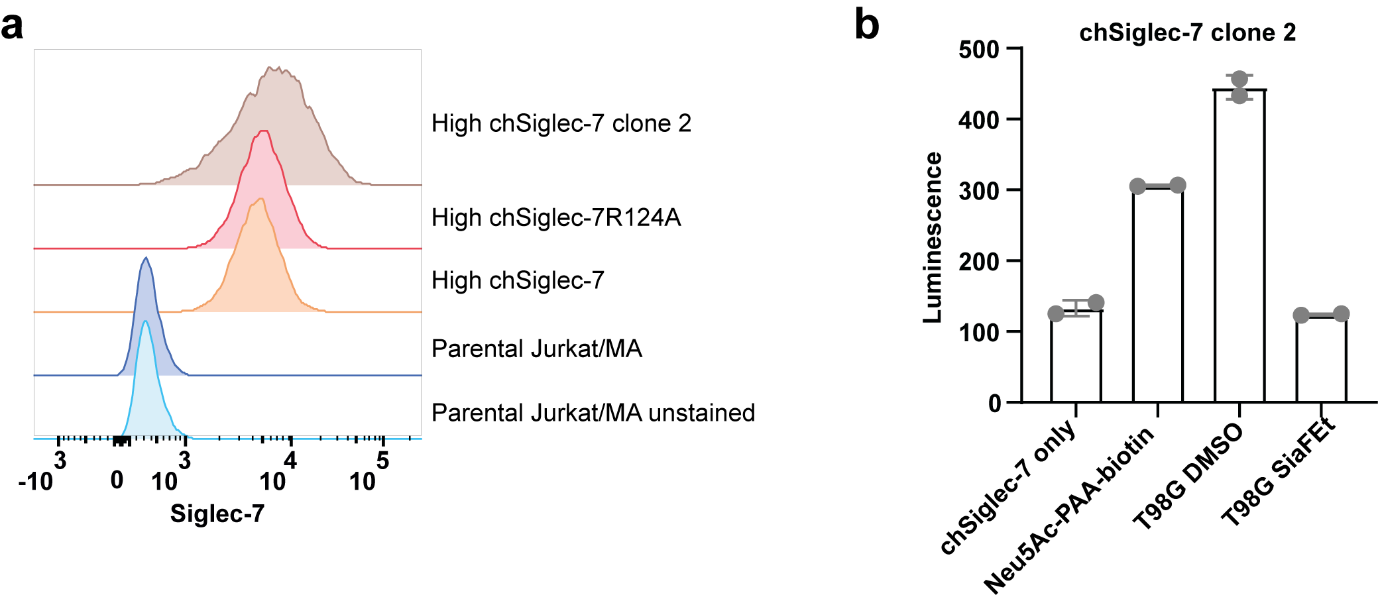


**Supplementary Fig. 3** chSiglec-7 expression and signaling by clone 2. **(a)** Siglec-7 expression by chSiglec-7 expressing Jurkat/MA, chSiglec-7R124A Jurkat/MA cells or chSiglec-7 clone 2 Jurkat/MA cells was determined by flow cytometry. **(b)** chSiglec-7 clone 2 Jurkat/MA cells were left unstimulated, cultured with Neu5Ac-PAA-biotin polymer or co-cultured with T98G cells (treated for 3 days with SiaFEt or DMSO). Luminescence was determined using a luciferase assay. Bar diagrams show mean ± SD.


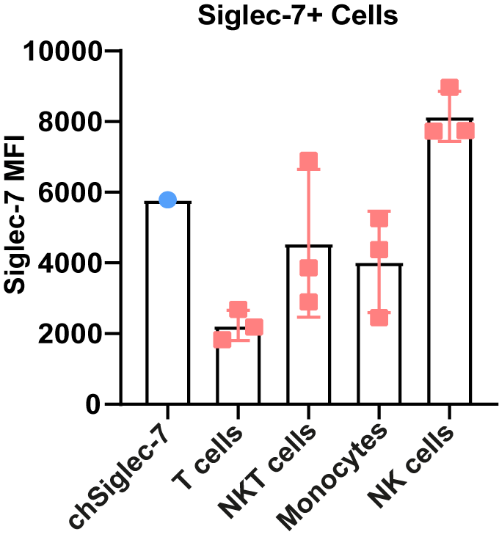


**Supplementary Fig. 4** comparison of chSiglec-7 levels on Jurkat/MA cells with endogenous Siglec-7 levels on immune cells. Immune cells were isolated from blood of 3 healthy donors and chSiglec-7 expression levels on Jurkat/MA cells and Siglec-7 expression levels on immune cells were determined by flow cytometry. Immune cells were gated based on CD3, CD14 and CD56 expression. Bar diagram shows mean ± SD.


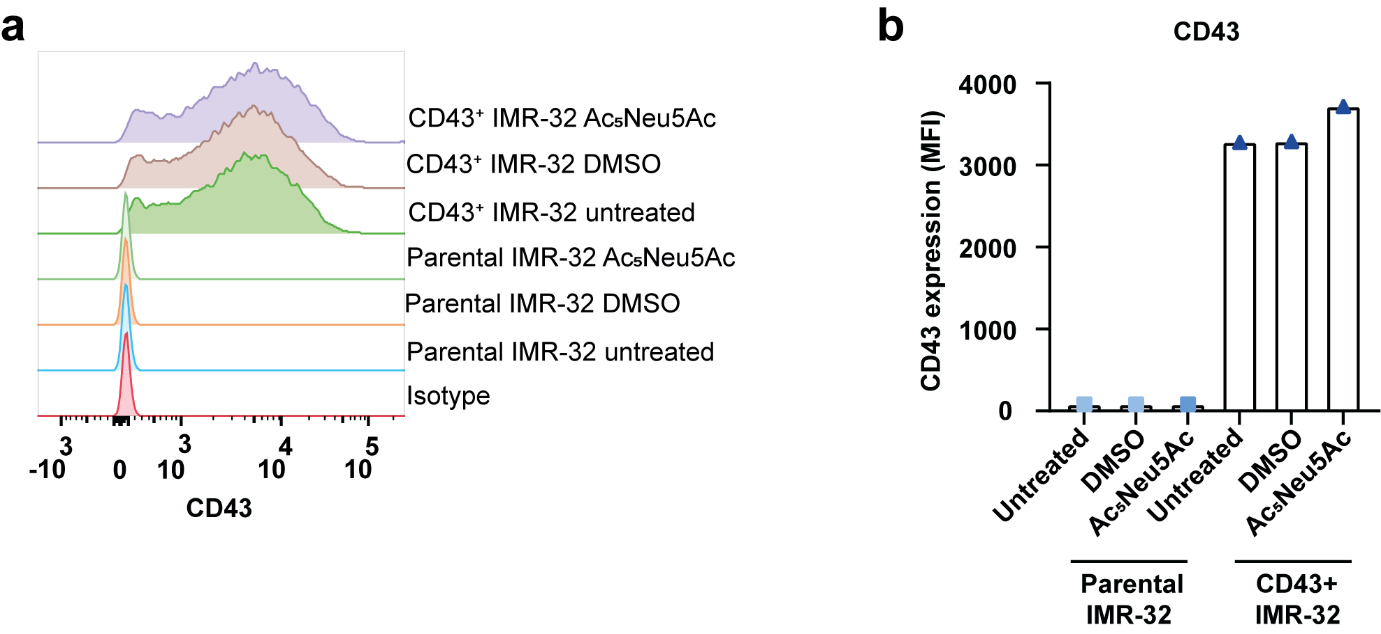


**Supplementary Fig. 5** CD43 expression of parental IMR-32 cells and sorted CD43+ IMR-32 cells. **(a)** CD43 expression by parental IMR-32 cells and by IMR-32 cells electroporated and sorted for CD43 expression was assessed by flow cytometry and (b) mean fluorescence intensity (MFI) was quantified (n=2). Cells were pretreated with Ac_5_Neu5Ac or DMSO as vehicle control for 3 days, and medium with treatment was refreshed on day 2. Representative data from two experiments are shown.
